# Big Data, Machine Learning, and Population Health: Predicting Cognitive Outcomes in Childhood

**DOI:** 10.1038/s41390-022-02137-1

**Published:** 2022-06-09

**Authors:** Andrea K. Bowe, Gordon Lightbody, Anthony Staines, Deirdre M. Murray

**Affiliations:** INFANT Research Centre, University College Cork, Ireland; INFANT Research Centre, University College Cork, Ireland; Department of Electrical and Electronic Engineering, University College Cork, Cork, Ireland; School of Nursing, Psychotherapy, and Community Health, Dublin City University, Dublin, Ireland; INFANT Research Centre, University College Cork, Cork, Ireland

## Abstract

The application of machine learning to address population health challenges has received much less attention than its application in the clinical setting. One such challenge is addressing disparities in early childhood cognitive development - a complex public health issue rooted in the social determinants of health, exacerbated by inequity, characterised by intergenerational transmission, and which will continue unabated without novel approaches to address it. Early life, the period of optimal neuroplasticity, presents a window of opportunity for early intervention to improve cognitive development. Unfortunately for many, this window will be missed, and intervention may never occur or occur only when overt signs of cognitive delay manifest. In this review we explore the potential value of machine learning (ML) and big data analysis in the early identification of children at risk for poor cognitive outcome, an area where there is an apparent dearth of research. We compare and contrast traditional statistical methods with ML approaches, provide examples of how ML has been used to date in the field of neurodevelopmental disorders, and present a discussion of the opportunities and risks associated with its use at a population level. The review concludes by highlighting potential directions for future research in this area.

## Early Life and Neurodevelopmental Plasticity

The phrase “the first 1000 days”, a period spanning conception to three years of age, is one increasingly seen in policy documents relating to child health.^1^ The phrase has its origins in the work of Professor David Barker, a physician and epidemiologist, whose trio of seminal articles published in the Lancet between 1986 and 1993 prompted the development of the ‘foetal origins hypothesis’.^2–5^ A fundamental concept in Barker’s work was developmental plasticity, formally defined as “the ability of a single genotype to produce more than one alternative form of structure, physiological state, or behaviour in response to environmental conditions”.^6^

It is widely accepted that early life is a unique period, differing from adulthood, during which the brain shows greater potentiality toward plasticity.^7^ This is explained in part by the highly dynamic dendritic spines, which are predominant during early development, and display huge potential for adaptability but, which over time and in adulthood are much more stable.^8^ Early life is the most crucial period for shaping the developing brain, and represents a window of both vulnerability and opportunity.^9^ A failure to achieve early foundational cognitive skills may result in a permanent loss of opportunity to achieve full cognitive potential. Conversely, with appropriate intervention, it is a period during which there is opportunity to disrupt the intergenerational transmission of inequity, particularly with regard to the developing brain.^9^

## Cognitive Function and The Importance of Social Determinants

Cognitive function is a broad construct consisting of multiple domains, which can differ across literature and disciplines. Most definitions consistently refer to domains of learning, understanding, reasoning, problem solving, memory, language, attention, and decision making.^10–12^ There has been considerable debate in the literature regarding the measurement of cognitive functioning, but also regarding its representativeness of overall human functioning.^13^ While a thorough discussion of this debate is beyond the scope of this article, it is important to clearly acknowledge that childhood cognitive function is only one domain of overall child development. Other domains, including motor development, the development of positive social skills and emotional intelligence, and the establishment of appropriate behaviours are both intimately intertwined and of commensurate importance.^14^

For many children, particularly those exposed to adverse environmental conditions in early life, the true potential of their cognitive ability will never be reached. This gap between potential and actual ability brings with it significant implications for outcomes across multiple domains throughout the life course, including educational attainment^15^, social mobility^16^, financial well-being^17^, and survival.^18^ Birth and pregnancy cohort studies have to date provided an evidence-base illustrating the important early life determinants of cognitive development. Consistently, socioenvironmental factors have been shown to be among the strongest determinants of cognitive function in childhood.^19–25^ The effect of socioenvironmental disadvantage on cognitive function can be seen as early as 2 years of age, but if unabated, the adverse effect of disadvantage on cognitive function appears to be amplified over time.^22^

## Evidence for Early Intervention

Across epidemiological, animal, and human research there is consensus agreement that early life is a period of fundamental importance in cognitive development, and strategies for intervention should ideally be targeting this unique period.^26–33^

One of the most widely cited examples supporting early cognitive intervention is the Abecedarian Study, a small randomised controlled trial begun in 1972, and involving infants at risk of poor cognitive outcomes based on an individual risk index which combined multiple factors including parental education, family income, welfare payments and the school history of family members.^34^ The trial involved 111 infants, of whom 57 were randomly assigned to an intensive preschool programme, which commenced at a mean age of 4.4 months. The intervention consisted of high quality early education delivered with high teacher to pupil ratios, five days per week for fifty weeks of the year, with free transportation and a focus on family involvement.^34,35^ At age 3, significantly higher IQ scores were observed in the intervention group, and these persisted into adulthood. At age 21, the intervention group still had significantly higher IQ scores and academic achievement scores, as well as significantly more years of education completed compared to the control group.^36^

This pre-emptive approach to early cognitive intervention may also benefit other high risk cohorts such as pre-term infants, who on average have childhood IQ scores approximately 10 points lower than their full-term peers.^37,38^ A Cochrane review examining the effects of early intervention provided in the first 12 months of life, for infants identified as at risk based on a gestational age <37 weeks or a birth weight <2,500 grams, concluded that early interventions can improve cognitive outcomes in infancy, with benefits persisting into preschool age.^39^

Early pre-emptive approaches targeting high risk individuals have also been shown to alter neurodevelopmental trajectories for other outcomes such as autism spectrum disorders (ASD). Whitehouse et al demonstrated, in a randomized clinical trial of infants with very early signs of potential ASD, that pre-emptive intervention commenced around the first year of life reduced the odds of ASD classification at age 3. ^40^

These studies illustrate the effectiveness of early pre-emptive intervention, commenced prior to overt signs of cognitive difficulty and at the time of optimal neuroplasticity. Outside of the controlled trial setting, real world programmes, such as the federally sponsored Early Head Start programme in the United States, demonstrate that at a population level such interventions are both feasible, achievable, and effective with appropriate resourcing.^41,42^ However, achieving optimal individual outcomes from early intervention is dependent on the accurate identification and involvement of infants and families who are likely to benefit most.

## Current Population-based Developmental Screening

Internationally, many countries rely on universal developmental screening programmes to identify children who may benefit from early intervention programmes, with the majority of developmental screening assessment being based on the presence of a delay in reaching developmental milestones.^43^ Population-based screening programmes based on the principle of universalism apply structured developmental screening, independent of pre-existing risk factors, to all children. While this approach has many merits, there are significant limitations. Firstly, the opportunity for pre-emptive intervention is lost as these infants are already behind their typically developing counterparts at the time of detection, with valuable opportunities in the period of optimal neuroplasticity being lost. Secondly, population based screening programmes depend on voluntary presentation of parents and children, and tend to exemplify the inverse care law in practice, whereby the most vulnerable and most at risk of developmental delay, are least likely to attend.^44–46^ Thirdly, such screening programmes do not provide a systematic, robust assessment of the environmental, social, and demographic factors, to which the infant will be exposed in early life which are the strongest and most consistent predictors of cognitive outcomes in childhood.^47,48^

The birth of a child is unique in many ways, not least because there is almost universal interaction between a mother and a healthcare professional. Therefore, identification of high risk mother-infant dyads in the perinatal period could provide a unique opportunity to engage families with early intervention programmes. Machine learning and artificial intelligence (AI), which has shown great promise in prediction across other fields of medicine, may have potential to improve population-based strategies for the early identification of children at risk of poor cognitive outcomes, and this deserves further exploration.

## Machine Learning and Predicting Risk

Adverse cognitive outcomes in childhood are complex, heterogenous, and result from highly interactive relationships between biological, environmental, and social factors, the mechanisms of which are often poorly understood. This real-world complexity, which may be difficult to model, particularly with more traditional statistical methods, may have to date acted as a deterrent for researchers investigating risk prediction tools for this purpose. However, there is now increasing potential to statistically model such complex interactions with machine learning. With the huge recent advances in both the quantity and quality of data available through large birth cohorts, electronic health records, imaging data, genomics, video, medical imaging, and electronic devices, the application of artificial intelligence and machine learning may assist in finding the optimal predictive patterns to enable early interventions. ^49^

The ‘exposome’ is a term used for the totality of life course environmental exposures from the prenatal period onwards. ^50^ Adopting this lens in evaluating the role of environmental factors may enable a more complete understanding of the exposures involved in childhood cognitive development. ^51,52^ Prospective birth cohorts and linked electronic medical records provide a unique opportunity for evaluating such exposures as they can capture a wealth of valuable information on prenatal, perinatal, and early childhood exposures. Following an individual from the intrauterine period or from birth enables a longitudinal assessment of early life exposures and later life outcomes.^53^ There has been an increase in both the number and the size of birth cohort studies in recent years, and in Europe alone more than 110 birth cohorts representing 27 different countries have been formed.^54^ Additionally, the capacity to collect large volumes of data, to biobank serum samples, to perform genomics, and to use technology such as wearable devices has resulted in an explosion in the quantity, complexity, and depth of data now collected in such cohorts, providing huge opportunity to utilise such data to advance both our understanding of the aetiology of disease, and our ability to predict outcomes. To truly harness the value of these cohorts and data sources, methods of data linkage and harmonisation require further research. Through amalgamating data sources, large data repositories could be created which would be particularly valuable for all fields of research, but especially for AI.

Further work is needed in this area to address the issues around data protection, consent, and data sharing.

Generally two classes of statistical model are recognised − those where the aim is to understand the causal pathways leading to a certain outcome, and those where the aim is to predict as accurately as possible a certain outcome.^55^ These two classes have much in common, and a tangled, but often constructive relationship.^56^ Statistical models such as those intending to investigate the causal pathways between early life determinants and cognitive function in childhood estimate the effect size between a preceding factor and the outcome, adjusted for the confounding factors in this relationship, based on a formal modelling process.^57^ The inclusion of additional variables in the model is based on previous evidence, changes in the size of the effect between the causative factor and the outcome, and goodness of fit. ^56,57^

Prediction models however combine multiple predictors based on their ability to predict the occurrence of a particular event in the future (prognostic model) or their ability to predict the presence of a particular health state (diagnostic model).^58^ Prediction model studies aim to develop and validate multivariable prediction models which combine individual predictors to predict an individual’s probability of either having or developing a particular outcome.^59^

Published prediction models examining the prediction of cognitive outcomes among general pediatric populations are scarce compared to the published literature on prediction models for high risk groups such as pre-term infants.^57^ A summary of three recently published multivariable models predicting cognitive outcomes in general pediatric populations is shown in Table 1.^20–22^

A major limitation in the traditional statistical regression methods used in these models is their reliance on strong assumptions, many of which are unrealistic in complex real word data. Traditional regression methods are often used in ways which assume that the effect of a predictor on the outcome increases or decreases uniformly throughout the range of the prediction (assumption of linearity), however in truth many relationships are non-linear. It is often assumed that the residuals (the difference between the observed value of the dependent variable and the model predicted value of the dependent variable) have a constant variance at each level of the predictor (assumption of homoscedasticity), and that the residuals have a normal distribution.^60^ Methods exist to deal with all of these issues, but may not be properly or commonly used. Further limitations are the constraints around interaction terms in traditional statistical models. Traditional models assume predictors affect the outcome in an additive manner unless otherwise specified. Interaction terms must be determined a-priori and entered into the model, a process not commonly performed despite a wealth of evidence demonstrating strong interactions between many of the predictors in the models, such as that between prematurity and maternal education, or between preterm brain injury and socioeconomic status.^61–63^

There is considerable debate regarding the true differences between traditional statistical approaches and machine learning, often compounded by the fact that many machine learning techniques, such as linear and logistic regression, are drawn from traditional statistical methods.^64^ While traditional statistical models and machine learning models may use similar means to reach their goal, the key difference tends to lie between the objectives of the models. Traditional statistical methods, while they can be used for prediction, tend to focus on inferring relationships between variables, while following a defined set of assumptions and rules based on the data to which the model is fitted.^65–67^ The purpose of ML is to make a repeatable prediction by learning from patterns within the data, without prior assumptions or rules governing the process.^66,67^ ML does not make inferences regarding the causal relationship between features and outcomes. Using designated training data, with a defined set of potential predictors and a defined outcome, a ML algorithm can learn a set of rules from the data which can then be applied to novel, unseen data to make a prediction on the outcome. ^68^ ML approaches can handle enormous quantities of data, and also can combine multiple different forms of data from multiple different sources including epidemiological data, imaging data, environmental and genomic data. ML techniques can efficiently deal with missing data, and driven by the data and generalisation performance, are adept at handling feature redundancy (collinearity) and feature selection.^68^

A major advantage of machine learning is the ability to handle large numbers of highly interactive predictors and to combine them in nonlinear relationships. With both the quantity and diversity of data growing, non-linear and interactive relationships within data are increasingly important and may be the key to improving predictive accuracy. ML techniques can, to a point, automatically uncover such interactive relationships in the data without the need for a priori specification of interactions.^69,70^ For the purposes of predicting cognitive development, this is likely to be important. It is often the case that the effect of one predictor will change given the value of another predictor. For example, the combined effect of adverse socioenvironmental factors and preterm brain injury results in significantly lower childhood IQ than either alone.^63^

It should not be assumed, however, that ML techniques will always result in improved predictive performance compared to traditional methods.^71^ This is likely to depend on the outcome being predicted, the number of features involved in training the prediction, the type and variability of the features, and the presence of interactions between features. The optimal predictive algorithm will depend on the predictive problem, and prior to moving to more complex predictive models, traditional methods with logistic and linear regression modelling should always be trialled and reported for comparison.^71^

## Applications of Machine Learning in Neurodevelopmental Pediatrics

The past decade has seen a rapid expansion in the application of ML and AI, for the purposes of diagnosis and prognosis, in clinical medicine.^68^ Significant progress has been made specifically in the areas of radiology and imaging, pathology, and cancer. ^72^ There is growing interest in the application of machine learning in the field of pediatric neurodevelopmental disorders (NDDs), a heterogenous group of disorders impacting different domains of function including cognition, language, social, motor and behavioural, but progress has been slower.^73^ The term NDD includes conditions such as intellectual disability, autism spectrum disorder (ASD), and attention deficit hyperactivity disorder (ADHD). ^73^

Diverse data, ranging from MRI data to motor skills data collected via drag-and-drop gaming devices, have been combined with machine learning techniques for both diagnostic and prognostic purposes in the area of NDDs. Examples are provided in Table 2. Accurate, objective, diagnostic tools could be a particularly valuable aid to clinicians in this field, where many of the conditions currently rely on subjective, labour intensive, expert clinical assessments. In many countries, this results in lengthy waiting lists for assessment in developmental pediatric clinics and delays in diagnosis for those affected.

One example is ADHD, the diagnosis of which relies upon a detailed clinical assessment based upon observed behaviour and reported symptoms.^74^ Pupillometric data (data based on dilation of the pupil) from 50 participants age 10-12 years used in a support vector machine (SVM) algorithm achieved a sensitivity of 0.773 and a specificity of 0.753 in the detection of ADHD, based on the results of nested 10-fold cross validation.^75^ This study however is limited by the small sample size of 50, and the absence of model evaluation on an independent test set raises questions over the generalisability of their model to wider populations. This is a limitation seen in many of the studies in this field.

MRI data has also been used in ML algorithms for the purpose of diagnosing ADHD. Sen et al trained a SVM using structural and functional MRI data from 558 participants. The final model, which produced an accuracy of 0.673, sensitivity of 0.454, and specificity of 0.851 on an independent test set of 171 participants, is unlikely to be clinically useful in its current form, but suggests there may be signals within such data that could assist in the diagnosis of ADHD.^76^

For ASD, another neurodevelopmental disorder which benefits from early intervention but for which diagnosis is often delayed, ML has been increasingly studied as a potential tool for early detection.^77^ A 2019 systematic review and meta-analysis examining the accuracy of ML algorithms based on brain MRI data for detecting ASD reported a pooled Area Under the Receiver Operating Curve (AUROC) of 0.90, a sensitivity of 0.83, and a specificity of 0.84 for ML models trained with structural MRI data. The results should however be interpreted with caution, as the majority of included studies performed internal validation only, and did not externally validate their models in independent test sets.^78^

While the examples outlined thus far have largely focussed on ML for diagnostic purposes, ML methods have also been deployed for prediction of later NDDs.^79,80^ A deep learning model, trained on white matter connectomes from brain MRI data obtained at time of birth, and tested on an independent test set, correctly classified 84% of infants as having below or above the median level of cognitive ability at 2 years of age.^80^ The results, which are based on a relatively small sample size of 37 in the independent test set, are promising but the clinical utility at a population-level is likely limited.

Novel methods of neurodevelopmental assessment, outside of traditional medical investigations, are also being analysed using ML algorithms. For example, a deep learning model trained on data from a touch-based computer game, aimed to harness this measure of motor skill to detect developmental disabilities in children. An AUROC of 0.817 based on internal cross-validation provides a basis for further research in this area.^81^

## Moving from Clinical to Population-based Prediction

Acknowledging the limitations of these studies, overall the results indicate that artificial intelligence and machine learning may have an important role in the early prediction of certain neurodevelopmental disorders. However, many of the prediction studies in this field to date are based on data, such as MRI and EEG, which is not routinely collected at birth at a population level. Therefore, in clinical practice these studies may provide methods of prediction for higher risk populations who undergo these investigations, but are likely to be largely unsuitable for implementation at a population-based level due to resource constraints and cost. At a population level, machine learning would appear to be more advantageous when used with data that is readily available, cost effective to collect, and available early in life. This however may come at the cost of reduced predictive ability compared with models based on direct physiological and anatomical measures.

In the context of population health, the application of machine learning to address population health issues and mitigate health disparities has received much less attention than its clinical applications. Prediction models at a population level tend to use existing electronic medical records, large cohort studies, environmental databases, government databases, and internet based data.^82^ Population based data sources have the potential to incorporate social, environmental, and other structural determinants in prediction models, potentially improving accuracy.^83^ Social determinants of health have long been studied in epidemiological research, but findings of these studies emphasise the need for methods that are better able to capture the complex, interactive, and flexible relationships between these predictors and health outcomes.^83^

The majority of well-established predictors of cognitive function in childhood, including poverty, gestational age, drug, alcohol and tobacco use during pregnancy, birthweight, admission to the neonatal intensive care unit, parental education and socioeconomic status, are predictors that are readily available in the perinatal period. To date, the accuracy of algorithms applying traditional statistical methods to such data for the purpose of identifying children at risk of low cognitive function in childhood have shown mixed results with regards to their accuracy (Table 1). The question of how we can best harness this information for identifying high risk children remains. Application of machine learning methods to large population based datasets, using readily available, routinely collected information may assist in this objective but remains relatively unexplored in the literature.

## Risks of Individual Risk Predictions and Machine Learning Algorithms

There are undoubtedly benefits to the early identification of children at risk of poor cognitive outcomes, namely providing early interventions during the period of optimal neuroplasticity and potentially altering the cognitive trajectory for the individual child. However, this approach is not without risk.

Early identification of those at risk is not synonymous with labelling, yet even the use of terms such as “at risk” or “higher risk” could have detrimental effects on both the child and their family. Labelling a child at a very early stage of life, as being at risk of a poor cognitive outcome, can perpetuate a self-fulfilling prophecy due to well documented effects on parent and teacher expectations, as well as the potentially detrimental effect on a child’s own self-concept.^84–86^ Furthermore, using a label based largely on social factors, which are transmitted through generations and rooted in inequities generated by social and political policy, may shift the blame of societal failures onto the individual child or family. These risks are especially pertinent for children and families who may be incorrectly identified as at risk on a screening or risk prediction tool and who are unlikely to benefit from early intervention. For those correctly identified, the question is whether these risks are outweighed by the risk of overlooking a child until their cognitive difficulties manifest in overt signs such as academic failures, a point at which intervention may be too late.^87^

There are also broader ethical considerations with the use of machine learning algorithms in healthcare in general.^88–90^ There is a growing body of literature examining algorithmic fairness, a topic warranting urgent attention following examples of ML algorithms exacerbating health inequities, raising serious ethical concerns.^91^ Each step of development of a ML algorithm is fraught with potential for bias - from the initial decision to use ML for the specific problem, to how it is deployed in practice.^92^ A major threat to algorithmic fairness is the inherent bias in the data on which many of these models are built. For example, healthcare utilisation data represents those who attend for healthcare, but not necessarily those who need it; cohort data tends not to represent marginalised populations such as the homeless who may be unable to participate in longitudinal follow up. A further threat is the use of “black box” models which lack transparency, cannot be interpreted by the user, and therefore have inherent risks for producing bias. There are opportunities to harness the power of ML in addressing population health challenges, but stringent assessment of potential for bias, quality, and applicability are required prior to dissemination in practice.^93^

## Conclusions and Future Research

There is an optimal window in early life during which the trajectory of cognitive development is more amenable to change.^94^ Infants at risk of poor cognitive outcomes due to prenatal, birth-related, or socioenvironmental risk factors, whom we fail to identify and provide with appropriate interventions, may never meet their true cognitive potential. For children who begin their educational journey lacking basic cognitive skills, a downward spiral of repeated failure, grade retention, and behavioural difficulties may culminate in school dropout, and its associated future implications which include unemployment and substance abuse.^95^ Equally it is the case that these children at higher risk are likely to live in poorer households, in more deprived areas, and be members of marginalised groups, so population level interventions including educational policy, planning policy, and economic policy, also have a central role to play.

It is estimated that less than half of children with developmental delay are detected prior to school entrance, with the vast majority of those detected receiving no intervention in the very early years.^96,97^ It is abundantly clear that the current practice of waiting for delays to present prior to intervention is inadequate and goes against the large body of evidence highlighting the optimal period of neuroplasticity in early life.^98^ The risk factors for poor cognitive outcomes in childhood are well established in the literature, are amenable to identification in the perinatal period, and are often routinely collected. Yet, to date we have been largely unable to harness this information in a personalised and meaningful way to identify the high-risk mother-infant dyads and provide appropriate interventions.

Our improved capacity to collect large volumes of rich data and to apply novel statistical methods particularly suited to prediction provide an opportunity for researchers and clinicians to investigate alternative methods of identifying, at an earlier stage, children at risk of developmental and cognitive delay. Utilising, to the best of their predictive ability, currently available data in the form of birth cohorts, electronic health records, and population registries should be a first step.

Addressing health and social inequity is an age old problem crying out for novel solutions. In this review we have focussed primarily on the domain of cognitive development. This is not to put undue emphasis on cognitive ability as a measure of success, nor to endorse a meritocratic society where a child’s emotional, social, or creative skills are viewed as less important. The purpose is to strive towards creating a society whereby every child is given the opportunity to meet their cognitive potential, irrespective of the circumstances into which they are born. To achieve this, high risk infants and their families should be supported with appropriate, evidence-based interventions at the earliest possible stage, prior to the onset of cognitive delay. This requires early, accurate identification of those who will benefit. Combining the strengths of machine learning, big data analysis, and pediatric public health for a potential solution deserves thorough exploration for the individual, community, economic, and societal benefits which could result.

## Figures and Tables

**Table 1:** Study Characteristics of Multivariable Prediction Models for Cognitive Outcomes in Childhood

Authors	Camargo-Figuera et al ^20^	Camacho et al^21^	Eriksen et al^22^
Journal	BMC Pediatrics	Paediatric Perinatal Epidemiology	PLOS One
Continent	South America	Europe	Europe
Sample	Pelotas Birth Cohort	Millennium Cohort Study	Lifestyle During Pregnancy Study (sampled from the Danish National Birth Cohort)
Design	Prospective cohort	Prospective Cohort	Prospective cohort
Sample size	3,312	9,487	1,782
Year of Recruitment	2004	2000-2002	1997-2003^a^
Exclusion Criteria	Conditions associated with very low IQ e.g. severe mental retardation	Nil	Multiple pregnancies, language barrier, impaired hearing or vision, congenital disabilities implying mental retardation
Age at Cognitive Assessment	6	3	5
Cognitive Assessment	Wechsler Primary and Preschool Scales of Intelligence -III	Bracken School Readiness Assessment	Wechsler Primary and Preschool Scales of Intelligence - Revised
Cognitive outcome variable	Binary. Low IQ defined by a z-score < −1.	Binary. Not school ready defined by score <1 standard deviation below mean	Continuous
Number of risk factors at outset	32	29	27
Rationale given for candidate variables	Yes - selected based on previous literature and availability	Yes − selected based on previous literature and availability	No − but broad range (>20) selected
Statistical Model	Multivariable logistic regression	Multivariable logistic regression	Multivariable linear regression
Method of initial screening of candidate variables	Forward and backward stepwise selection	Forward and backward stepwise selection	Univariable association p ≥ 0.10
Interaction terms fitted	No	No	No
Multicollinearity addressed/discussed	No	Yes	Yes
No. Predictors in Final Model	13	13	9
Validation performed	Yes. Internal and external validation performed.	Yes. Internal validation only.	No
Predictive value measured	External validation Area Under Receiver Operating Curve (AUROC) 0.75 Sensitivity 70.3% Specificity 68%	Internal validation AUROC 0.80 Sensitivity 72% Specificity 74%.	R squared 0.29
Predictors in Final Model	Child − gender, height-for-age deficit; head circumference-for-age deficit Parental − breastfeeding, parental smoking, maternal perception of child’s health, skin colour Socioenvironmental − parental employment status, maternal education, income, number of siblings, number of persons per room	Child − gender, ethnicity, developmental milestones Parental − maternal age, maternal mental health, breastfeeding Socioenvironmental − socioeconomic class, maternal education, income, number of children, employment status, housing type	Child - gender, birth weight, height, head circumference Parental − maternal BMI, breastfeeding Socioenvironmental − Maternal IQ, Parental education

a In 2003, a prospective follow-up of 1750 mother-child pairs was initiated, sampled on the basis of maternal alcohol drinking patterns from The Danish National Birth Cohort (DNBC)

**Table 2:** Examples of Machine Learning Methods Applied in Neurodevelopmental Paediatrics

**Authors**	Das and Khanna ^75^	Sen et al ^76^	Emerson et al ^79^	Girault et al ^80^	Kim et al ^81^
**Year Published**	2019	2018	2017	2019	2021
**Purpose**	Diagnostic	Diagnostic	Prognostic	Prognostic	Diagnostic
**Outcome**	ADHD	ADHD	ASD at 24-months of age	Cognitive ability at age 2 years	Developmental disability
**Outcome Measure**	Diagnosed by child neurologist using DSM-IV criteria	Diagnostic criteria differed across study sites	DSM-IV Criteria	Mullens Scales of Early Ability. Outcome categorised as above or below the median	Diagnosis made by paediatric psychologist
**Features**	Pupillometric data	Structual and functional MRI	MRI at 6-months of age	MRI at birth	Drag and drop data from computer based game
**Participants**	50 (28 cases, 22 controls)	558 (279 cases, 279 controls)	59 infants with high familial risk of ASD (11 cases, 48 controls)	115 infants	370 children (147 cases, 233 controls)
**Algorithms trained**	LR, SVM, KNN, NB, DT, RF	SVM	SVM	DL	DL
**Evaluation method**	Nested 10-fold cross validation	Independent test set (77 cases, 94 controls)	Cross-validation with nested leave one out procedure	Independent test set (22 cases, 15 controls)	10-fold cross validation
**Evaluation Result (best performing models)**	NB: AUROC 0.871, sensitivity 0.656, specificity 0.863;	Accuracy 0.6725, sensitivity 0.4545, specificity 0.8510	Accuracy 0.966, sensitivity 0.818, specificity 1.000	Accuracy 0.838, Sensitivity 0.864, Specificity 0.800	AUROC 0.817, Sensitivity 0.757, Specificity 0.740

LR − Logistic Regression, SVM − Support Vector Machine, KNN − K-Nearest Neighbors, NB − Naïve Bayes, DT − Decision Tree, RF- Random Forest, ADHD − Attention Deficit Hyperactivity Disorder, ASD − Autism Spectrum Disorder, DSM-IV − Diagnostic and Statistics Manual of Mental Disorders

## Impact

- To date, the application of machine learning to address population health challenges in pediatrics lags behind other clinical applications. This review provides an overview of the public health challenge we face in addressing disparities in childhood cognitive development and focuses on the cornerstone of early intervention
- Recent advances in our ability to collect large volumes of data, and in analytic capabilities, provide a potential opportunity to improve current practices in this field
- This review explores the potential role for machine learning and big data analysis in the early identification of children at risk for poor cognitive outcomes.
